# CO_2_ and SO_2_ Capture by Cryptophane-111 Porous Liquid: Insights from Molecular Dynamics Simulations and Computational Chemistry

**DOI:** 10.3390/nano15080616

**Published:** 2025-04-17

**Authors:** Pablo Collado, Manuel M. Piñeiro, Martín Pérez-Rodríguez

**Affiliations:** 1 Departamento de Física Aplicada, Universidade de Vigo, E36310 Vigo, Spain; pcollado@iqf.csic.es; 2 Instituto de Química Física Blas Cabrera (IQF), (CSIC), C/Serrano 119, 28006 Madrid, Spain; 3 Modelización y Simulacón de Materiales Nanoestructurados, Universidade de Vigo, Unidad Asociada al CSIC por el IQF, Campus Lagoas-Marcosende, 36310 Vigo, Spain

**Keywords:** porous liquid, SO_2_, cryptophane-111, CO_2_, molecular dynamics, DFT, absorption, capture, separation

## Abstract

A computational study of the encapsulation of a gaseous mixture of CO_2_ and SO_2_ in a Type II porous liquid is performed under different conditions. The system is composed of cryptophane-111 molecules dispersed in dichloromethane, and it is described using classic molecular dynamics at atomistic resolution. Gaseous CO_2_ tends to almost fully occupy cryptophane-111’s cavities during the first phases of simulation, and, afterwards, it is surpassed by SO_2_’s tendency for occupation. Calculations are performed at five different temperatures in the range of 273 K–310 K, finding a positive correlation between SO_2_ adsorption and temperature. An evaluation of the radial distribution function of SO_2_ and CO_2_ with respect to cryptophane-111 is employed to quantify the number of captured molecules, and an energy study using Density Functional Theory methods is also performed to evaluate the relative stability of the two gases inside the porous liquid.

## 1. Introduction

Global industrialization has led to a rapid increase in the atmospheric greenhouse gas concentration and the release of other environmentally harmful compounds that have contributed to a set of global transformations [1,2,3,4]. With the aim of countering these emissions, different methods of capture have been developed over the years, especially for CO_2_ emissions [5]. Porous liquids (referred to as PLs hereafter for brevity) are an emerging material with high potential in gas capture/separation areas [6].

PLs were first described by O’Reilly et al. [7] as fluid media with permanent porosity. Firstly classified into three groups, namely, Type I, Type II, and Type III [8], recently, a new group, Type IV, was proposed by Bennet et al. [9].

Type I PLs are composed of liquids, where each individual molecule presents a permanent and rigid cavity, so they cannot be automatically filled, making them hard to synthesize because of the need for this neat state. They present high melting points and a tendency to crystallize outside certain ideal range conditions [10]. Adding flexible alkyl chains reduces their porosity, as well as their melting point [11]. Type II PLs are composed of rigid cage molecules and a sterically hindered solvent, which cannot enter the cage cavities. Type III PLs consist of Metal Organic Frameworks (MOFs) suspended in a sterically hindered solvent. The latter are easier to synthesize and eliminate the need for low melting points or high solubilities because of them being in suspension. However, they may present phase separation and generate precipitation due to the different natures of the pores and their distribution on the MOF, lowering the overall porosity. To avoid this, some studies have considered the use of nanoparticles or nanocrystals despite their large size and the consequent stability issues [12]. Finally, Type IV PLs are defined as MOF species with intrinsic porosity isolated in liquid form, meaning that they do not need a solvent for their suspension. So far, few studies have been carried out on PLs, and even less on gas absorption. Several studies have suggested the possibility of developing a renewable route to adsorb CO_2_ under mild conditions [13,14,15,16,17,18,19,20,21,22]. However, there is still limited knowledge about the adsorption of other greenhouse gases in porous liquids. As examples, we can cite the work of Oltean et al. [23], where the CH_4_ interaction with cryptophane-111 (C-111) in the presence of H_2_ is described, or the contribution by Dou et al. [24], where SO_2_ absorption/desorption is analyzed. In this study, using molecular dynamics (MD) and electronic Density Functional Theory (DFT) calculations, we analyze the process of the encapsulation of a SO_2_ and CO_2_ mixture in a Type II PL at different temperatures, deepening the understanding of the competitive process behind the encapsulation and the thermodynamics. The PL consists of C-111 as the porous molecule and dichloromethane (DCM) as the solvent. In previous studies, we also analyzed this PL when studying the encapsulation of H_2_, SO_2_, and CO_2_ [25,26]. C-111 is the smallest molecule from the cryptophane family, and it was first analyzed as a possible gas collector by Fogarty et al. [27]. Since then, it has been intensively studied for its remarkable complexation with Xe [27,28,29].

Cryptophanes are aromatic molecules, characterized by three symmetric folds of cyclotribencylene in a crown conformation, defining an inner cavity with a stable geometry. Because of stereoisomerism, they present a great variety of structures and properties, one of which is the possibility of enclathrating a guest molecule in their cavity, and the nature of their bonded parts and functional groups define the size and flexibility of the cage geometry [30]. The narrow inner cavity of C-111 makes it a suitable candidate molecule to study the encapsulation/separation of small gas molecules. Because of that, we selected SO_2_ and CO_2_ as guest molecules in view of their size, which is comparable to that of Xe, making their adsorption feasible. As for the solvent, DCM was selected due to the solubility of C-111. The inner volume of the cavity of C-111 molecules ranges from 32 Å^3^ to 72 Å^3^ in the most expanded state, and, despite DCM presenting a molecular volume within that interval, it cannot pass through the entrance of the cavity, as discussed by Buffeteau et al. [31]. CO_2_ is known for being quite soluble in DCM [32,33], and SO_2_ is commonly commercially presented dissolved in DCM, so their mutual solubility is not an issue.

The choice of this particular Type II porous liquid was motivated by various reasons, including the previous results and performance demonstrated by C-111 in our work [25,26], as well as the accessible simulation CPU times for this type of PL. Because of the environmental and overall purposes of this kind of material, we worked with atmospheric pressure and a range of different temperatures close to standard room temperature, avoiding the possible degradation of C-111 and the phase transitions of the gases. Additionally, our research group has performed molecular simulations to characterize gas diffusion through nanoporous structured solid materials, such as hydrates or clathrates [34,35,36,37,38,39], and the process of gas capture on PLs presents many similarities to these inclusion solids. The capture of the mixture of SO_2_ and CO_2_ has been recently studied using different approaches [40,41,42,43,44,45]. Considering an alternative for this study of other PLs, different issues have been taken into account: Type III PLs are bound for precipitation occurrence, possibly complicating the search for a viable option; Type I PLs present very complex molecular structures, making molecular simulations less accessible; and references available for Type IV PLs are still very scarce. Because of this, Type II PLs represent probably the most accessible option to currently approach this problem. In conclusion, based on the results of our previous works, we intend to achieve a better understanding of this kind of encapsulation with the use of comparative and qualitative explanations. With these studies, we aim to contribute to the theoretical understanding of potential alternatives to atmospheric greenhouse gas separation and capture, exploring the viability and efficiency of PLs in this context.

## 2. Computational Methods

We used the C-111, CO_2_, SO_2_, and DCM molecular topologies described in our previous works [25,26]. C-111’s atomic coordinates were retrieved from Joseph et al. [28], who studied the encapsulation of Xenon, and the corresponding topology was built using the Automated Topology Builder (ATB) v.3.0 repository [46]. The molecular model for DCM was also obtained through ATB from the work by Stroel et al. [47]. SO_2_ is a widely studied molecule, so examples of its topology were readily available at ATB, and we selected the “MOLID 370654” model. ATB uses a knowledge-based approach, in combination with QM calculations, to assign force-field parameters, and its results are known to be reliable [48]. A submitted molecule is initially optimized at the HF/STO-3G (or AM1 or PM3) level of theory, after which an initial draft output is available. Molecules with <50 atoms are then re-optimized at the B3LYP/6-31G* level of theory with the PCM implicit solvent model for water. The QM electrostatic potential (ESP) is then calculated from the B3LYP-optimized structure, which the ATB uses to obtain ESP-fitted charges. Finally, the QM Hessian is calculated for molecules with <40 atoms to improve the assignment of bond and angle terms.

Many CO_2_ molecular models developed for molecular simulation calculations are available in the literature. We decided to use the TraPPE [49] molecular model because of its widely reported excellent performance [50]. In previous studies, we used this CO_2_ molecular model in the study of solid–liquid equilibrium [51] and the description of CO_2_ hydrates [52] or CO_2_ organic clathrates [35], in all cases yielding excellent performance in systems including solid phases, essential to the present study.

All MD simulations were performed using GROMACS v.2021.5 software [53,54,55,56,57,58,59,60]. A typical calculation consisted of 100 ns of simulation with a 1 fs time step, except for when indicated otherwise. An initial energy minimization was performed using the conjugated gradient method to avoid molecular overlapping. Electrostatic long-range corrections were handled using the Particle Mesh Ewald (PME) technique [61], a 4th order of calculation, and a Fourier spacing of 0.1 nm. The dispersive van der Waals cut-off radius was set to 1 nm, and long-range dispersive corrections were applied to energy and pressure. A Nose–Hoover [62,63] thermostat was used to keep the temperature constant, with a 2 ps coupling constant, and a Parrinello–Rahman [64,65] barostat was used to set the pressure value, with a 4 ps coupling constant. All simulations were initiated by creating a bubble of the gas mixture in the simulation box, generated through the random insertion of C-111 and DCM molecules. Their final configurations were analyzed using GROMACS v.2021.5 tools and VMD v.1.9.4a51open-source software [66,67].

Fifteen molecular simulation setups were studied. The simulation boxes contained 14 C-111 molecules, 28 CO_2_ and SO_2_ molecules, and 600 DCM molecules.

Because DCM’s volume is smaller than the maximum cavity volume of C-111, special care was taken during the initial setup simulation to avoid the accidental insertion of a DCM molecule inside a C-111 cavity. During the simulation, DCM itself could not spontaneously access the cavity of C-111 because the cavity threshold was smaller than the average DCM molecule diameter.

After the energy minimization step, NpT molecular dynamics was performed at 1 bar and temperatures of 273 K, 283 K, 293 K, 300 K, and 310 K, maximizing the working temperature range between the boiling points of DCM and SO_2_. We built three different replica boxes for each setup and calculated the radial distribution functions for every temperature.

For the computation of the complexation energies, we employed four different DFT methods, as implemented in Gaussian 16, Revision C.01 [68], over individual molecules. We initially considered two well-known and reliable hybrid methods, suitable for the molecules tested: PBEh1PBE [69] and B3LYP [70,71,72,73]. Furthermore, due to the expected interaction type between the porous liquid and the captured gases, we used two additional functionals incorporating empirical dispersion terms: wB97XD [74,75] and the empirically corrected B3LYP, B3LYP-D3 [76]. All DFT calculations were performed using the 6-311+G(d) basis set, whose results are considered accurate for this kind of study [77], at an affordable computational cost. Indeed, a larger basis set did not provide significantly better results, being notably more expensive in terms of computational resources.

## 3. Results and Discussion

### 3.1. Simulation Analysis

**Figure 1:**
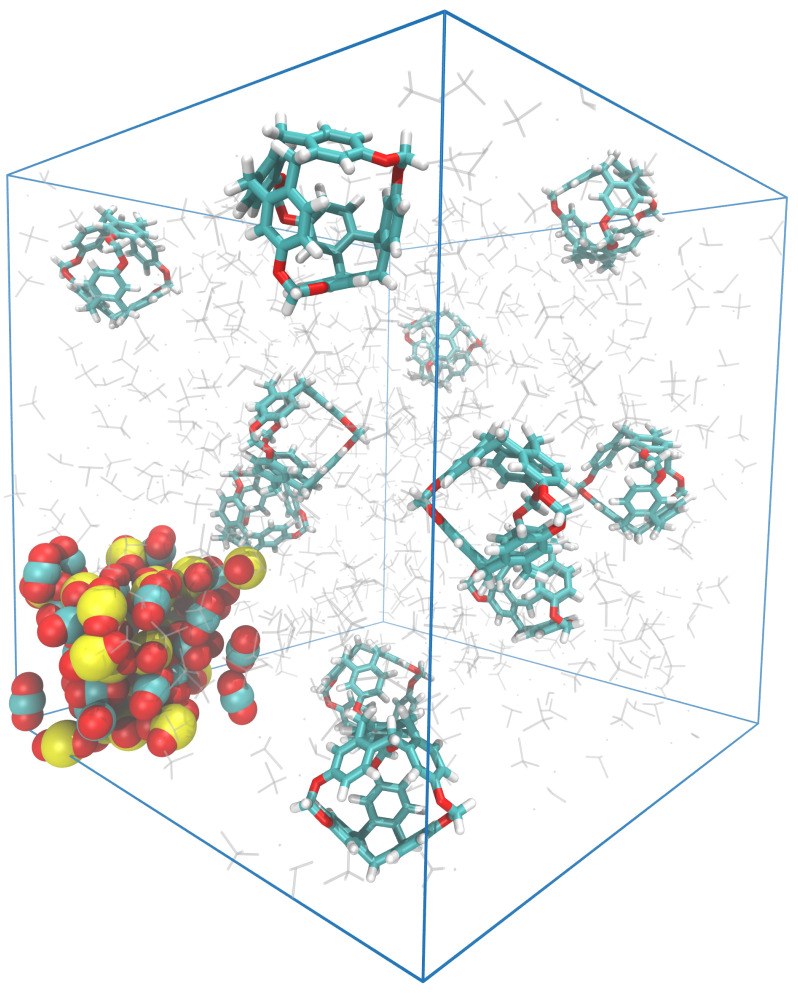
Snapshot of the initial configuration, showing the porous liquid (C-111 in DCM solvent) and gas bubble (CO_2_ and SO_2_) in the bottom-left part of the figure. SO_2_ is represented by yellow and red; CO_2_ is represented by blue and red.

Using the specifications mentioned before, simulation boxes were built with C-111 molecules, with CO_2_ and SO_2_ gaseous molecules in a DCM solution. We placed the CO_2_ and SO_2_ mixture in a bubble, trying to mimic the flow of a flue stream through the PL, as shown in Figure 1.

**Figure 2:**
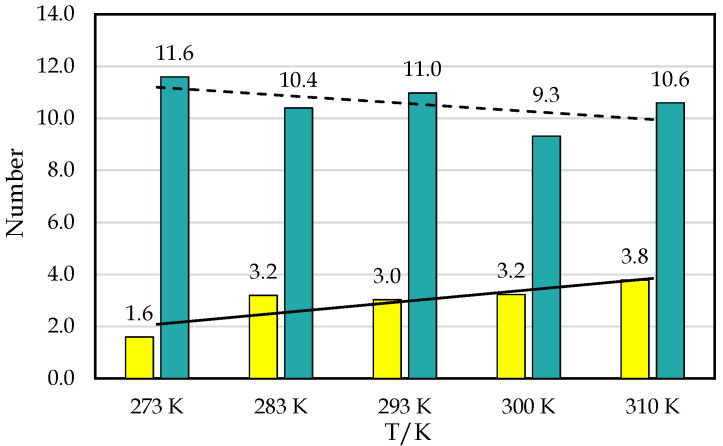
Number of occupied C-111 pores vs. T for CO_2_ (green-blue bars) and SO_2_ (yellow bars). As a guide, a dashed line is included for CO_2_, and a solid one is included for SO_2_.

**Figure 3:**
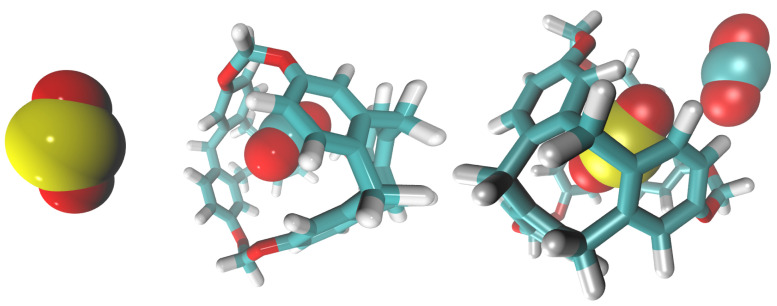
Snapshots of (**left** side) CO_2_ inside C-111 with an outsider CO_2_ and (**right** side) SO_2_ inside C-111 interacting with an outsider CO_2_. Elements are colored as follows: S, yellow; O, red; C, blue; and H, white.

We built three replicas of the system at each of the five different temperatures. After the simulations finished, we analyzed the production trajectory using Gromacs tools and VMD. We obtained the C-111 occupancy rates of CO_2_ and SO_2_ at each temperature, observing the behavior of both gases with C-111 throughout the simulation and examining how and how many interactions occur with C-111. We observed a greater selectivity towards CO_2_, as expected from our previous studies [25,26]. However, this selectivity towards SO_2_ decreased as the temperature increased. In Figure 2, we can observe an increase in SO_2_ adsorption rates, contrary to the CO_2_ trend. SO_2_ adsorption was more than two times larger at the highest temperature in the range tested, while CO_2_ suffered a continuous decrease. By carefully examining the simulation trajectories, we observed different events. At 310 K, CO_2_ encapsulated in a CO_2_/C-111 complex was substituted by SO_2_. CO_2_ was captured at 12.12 ns of the simulation and was pushed at 98.4 ns. This event resembles one in our previous work [25], where a CO_2_ molecule pushed another CO_2_ molecule. This event occurred at 300 K, meaning that this oddity could be improved at higher temperatures. However, SO_2_ molecules, when adsorbed, were never observed to be replaced by the SO_2_ or CO_2_ molecules present. In Figure 3, we can observe an example of these events, with the approximation of a CO_2_ molecule to an encapsulated SO_2_. This CO_2_ seemed to slightly attract SO_2_, with no other effect observed other than a little reorientation. In some cases, CO_2_ and SO_2_ molecules were observed to approach the C-111 cavity with the preferential orientation to obtain access, and, in these cases, SO_2_ was always the one captured. We also noticed an increase in the SO_2_ adsorption rates in the final simulation stages, as more CO_2_ was being desorbed. This suggests that SO_2_ could be favored thermodynamically over CO_2_, but its size makes it less likely to access the pore first.

**Figure 4:**
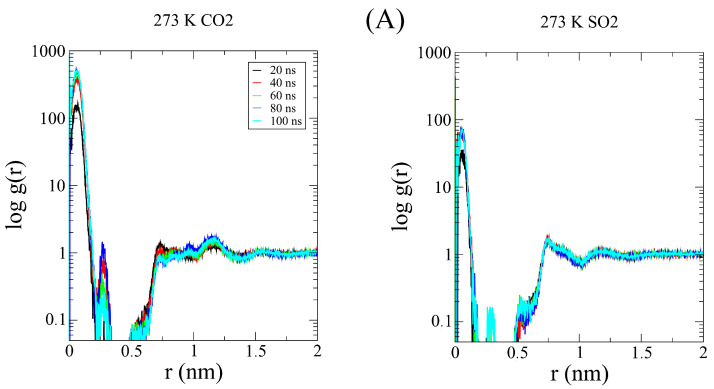

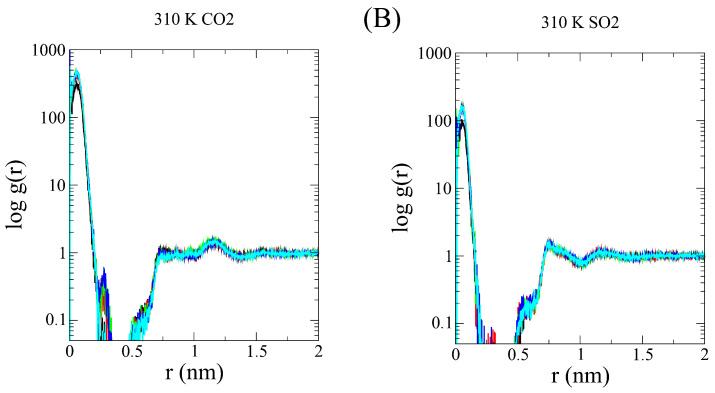
RDF of (**A**) CO_2_ vs. C-111 at 273 K and 310 K; (**B**) SO_2_ vs. C-111 at 273 K and 310 K.

**Figure 5:**
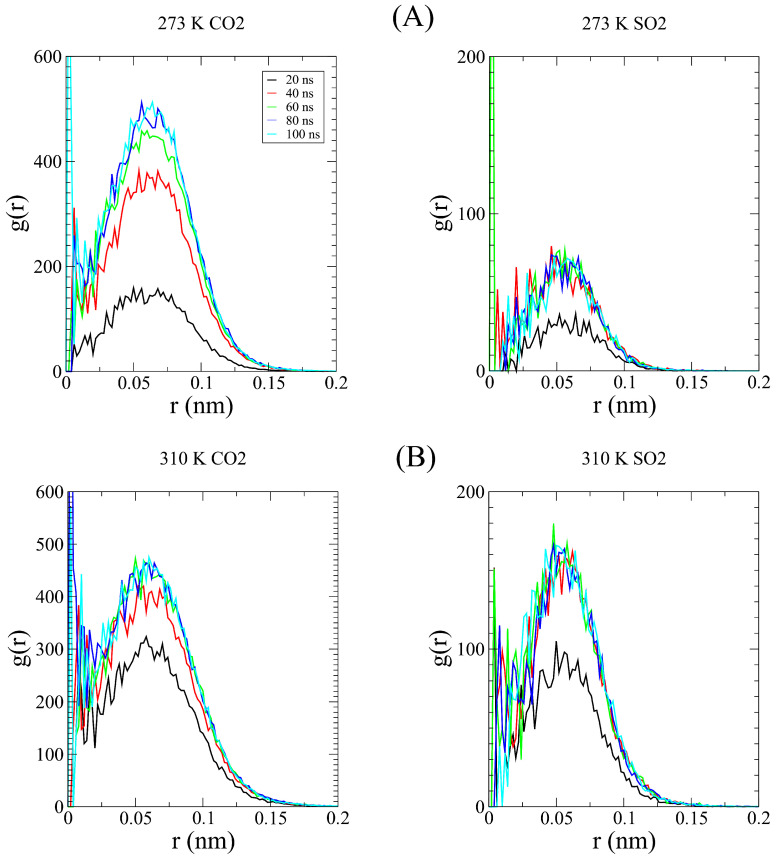
Adsorbed molecule RDF for (**A**) CO_2_ vs. C-111 at 273 K and 310 K; (**B**) SO_2_ vs. C-111 at 273 K and 310 K.

**Figure 6:**
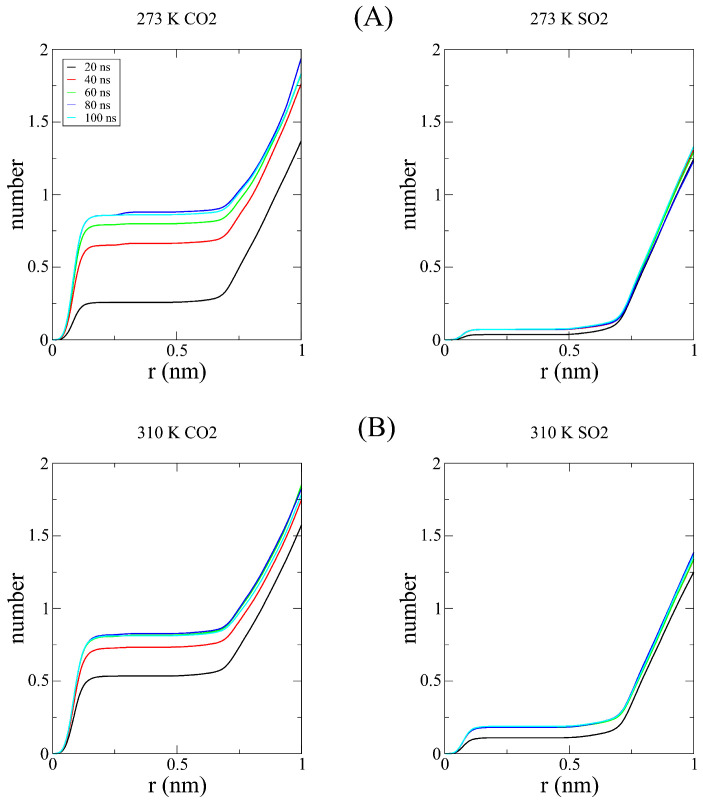
Mulative Sum RDF of (**A**) CO_2_ vs. C-111 at 273 K and 310 K; (**B**) SO_2_ vs. C-111 at 273 K and 310 K.

**Figure 7:**
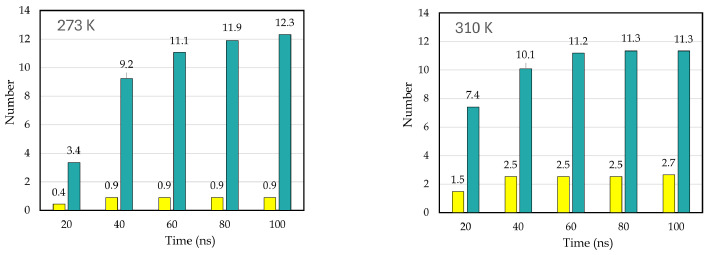
Number of occupied C-111 pores (out of 14) vs. time for CO_2_ (green-blue bars) and SO_2_ (yellow bars) in the base of the Cumulative RDF Sum of both temperatures.

The observed result can be interpreted as follows: As the temperatures increases, the SO_2_ kinetic barrier is lowered enough to begin compensating for the kinetic preference of smaller molecules such as CO_2_, prioritizing a higher proportion of SO_2_ being captured despite there being more CO_2_ in the pore. With the aim of checking this assumption, we performed a series of radial distribution function calculations, comparing both gases individually with C-111 as a reference. In Figure 4, the computed RDFs for adsorption are shown, with a logarithmic scale on the Y axis for clarity. As a reference, we used the center of mass of both molecules to represent the RDFs. The physical barrier (C-111 cage) was placed at 0.23 nm, as shown in the plot minima. However, for CO_2_, we obtained non-zero values at this distance. As discussed, adsorbed CO_2_ can be displaced by another CO_2_ or SO_2_ molecule. Because of this, a CO_2_ molecule can partially enter the C-111 cage while another CO_2_ molecule is already in there, explaining the presence of these non-negligible values. This phenomenon does not occur with SO_2_, which is not observed to leave the pore once captured. In Figure 5, we can observe this tendency of CO_2_ to be encapsulated since the first stages of the simulation and how SO_2_ starts to appear inside C-111 right after the initial 50 ns of the simulation have passed. We can also observe the increased tendency for the encapsulation of SO_2_ in the 310 K image while the profile of entering later than CO_2_ is still maintained. We performed Cumulative Sum RDF calculations, searching for a quantitative method to follow the capture process through the simulation. These Cumulative Sum RDFs represent the increase in pore occupation by the indicated molecule against time, with y=1 being the full occupancy of every C-111 molecule present in the simulation box. These results are shown in Figure 6. In Figure 7, we can observe the difference represented in the Cumulative Sum RDFs in terms of occupation numbers vs. time, showing how there is no linear correlation between occupation and time. In these figures, a greater difference in capture for CO_2_ at the lowest temperature is evidenced, reaching the maximum occupation in the middle stages of the simulation. For SO_2_, we observe low occupancy rates at the beginning of the simulation; however, at 310 K, this occupation duplicates, thus reducing CO_2_ capture.

**Figure 8:**
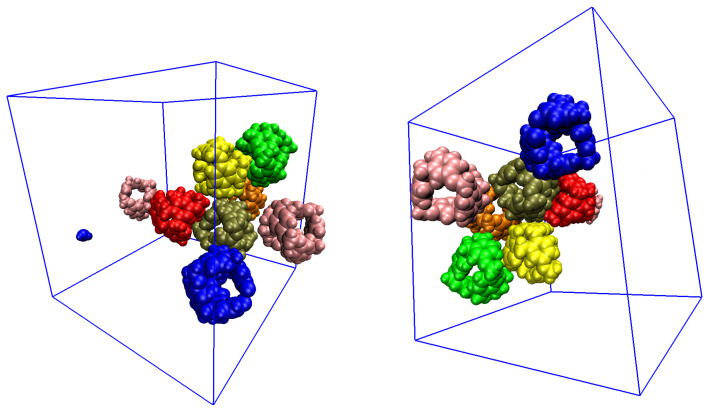
C-111 clusters during simulation.

Taking into consideration the possibility of C-111 precipitation, which occurs in Type III PLs, we decided to search for aggregates that could influence the capture process. Several clusters of C-111 formed occasionally during the simulations. We then expected some kind of interaction between the C-111 molecules, but the overall absorption rates did not seem to be affected because neither absorption nor desorption occurred just before, after, or during this cluster formation, and redispersion occurred, as shown in Figure 8.

### 3.2. DFT Calculations and Stability

In order to analyze the relative stability of the adsorbed species, we performed additional DFT simulations. Through these, we obtained the energies of C-111 and the adsorbed gases individually, as well as the energies of the resultant encapsulated complex. Afterwards, we subtracted the energies of the component molecules from the corresponding complexes, $\Delta E_{C\text{-}111[guest]}=E_{C\text{-}111[guest]}-(E_{C\text{-}111}+E_{guest})$, to directly obtain the interaction energies. We did not independently consider the deformation energies of the molecules, going from the free state to the complex. Other significant contributions, such as zero-point, thermal, or solvation energies, were also not taken into account. The reason for this is that, in the present study, our aims were mainly to check the validity of the conclusions extracted from the MD simulations and to establish the binding preferences for C-111, but not to obtain a precise value of those energies. However, we were interested in the influence that the dispersive interactions may have on the relative stability; therefore, we considered methods without (PBE and B3LYP) and with dispersion corrections (wB97XD and B3LYP-D3) to check this point, as mentioned in Section 2.

**Figure 9:**
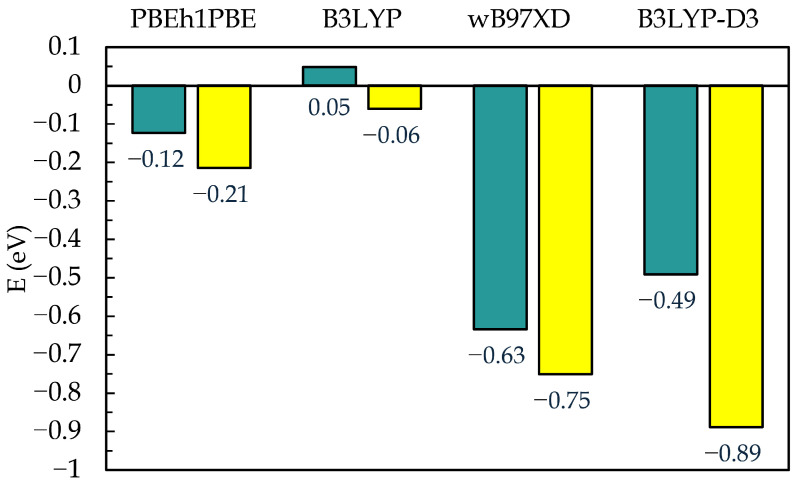
Binding energy in eV of the gas-C-111 complexes, $\Delta E_{C\text{-}111[SO_{2}]}$ (yellow bars) and $\Delta E_{C\text{-}111[CO_{2}]}$ (green-blue bars), obtained from different DFT methods.

The DFT results confirm what the MD simulations suggested, with SO_2_ being more stable than CO_2_ within the C-111 cavity in terms of energy, as shown in Figure 9. Each method represents both pairs as thermodynamically stable, except for C-111[CO_2_] in B3LYP, which yields a very low positive value. B3LYP shows the highest difference in terms of energy among the four methods used. The use of dispersion greatly increases the stabilization energy of both complexes, consistently keeping C-111[SO_2_] as the most stable. In B3LYP-D3, a significant difference between energies is observed in comparison with the wB97XD method, which perhaps can be attributed to the increased complexity of the B3LYP-D3 method [78]. We find many similarities in terms of energy in pairs (for all models, either including dispersion or not). Moreover, in the case of the global profile, the trend is coincident for all methods: $\Delta E_{C\text{-}111[SO_{2}]}$ is more stable for every method than $\Delta E_{C\text{-}111[CO_{2}]}$. If we perform the subtraction of $\Delta E_{C\text{-}111[SO_{2}]}$ minus $\Delta E_{C\text{-}111[CO_{2}]}$, we obtain almost identical differences of −0.09 eV, −0.11 eV, and −0.12 eV for PBE, B3LYP (non-dispersive), and wB97XD. However, the B3LYP-D3 method gives a result of −0.40 eV. All of these results point to C-111[SO_2_] as being the most stable complex, supporting the conclusions drawn from molecular dynamics, with a thermodynamical preference of C-111 for encapsulating SO_2_ rather than CO_2_, with the latter being favored by kinetics. This kinetic effect is supported by the relative size of both molecules, with CO_2_ being smaller and less sterically hindered to access the pore. The chemical difference between both molecules has to also be pointed out. With both consisting of a central molecule bonded to two oxygen molecules, CO_2_ presents a linear geometry ($\hat{OCO}=$ 180°), while SO_2_ has an angular one ($\hat{OSO}=$ 119°). Because of this, CO_2_ has no dipole moment but only a quadrupolar moment, while SO_2_ presents a significant dipole moment of 1633 D [79], close to the water value. In addition, due to the angular geometry, SO_2_ is effectively wider than it would be as a linear molecule. The differences in the overall geometry and dipole moment of CO_2_ and SO_2_ explain the easier encapsulation of CO_2_ and the greater stability of C-111[SO_2_] complexes.

### 3.3. Future Approaches

We discussed the viability of CO_2_ and SO_2_ encapsulation in Type II porous liquids, as well as feasible methods to improve the selectivity and efficiency of SO_2_ capture vs. CO_2_. However, further tests must be carried out to identify an efficient way to desorb these gases from C-111, achieving a renewable source of CO_2_ and SO_2_ recovery. We expect an easier desorption of CO_2_, as it has been shown to be able to leave the C-111 cavity by being pushed by another molecule. However, SO_2_ seems to be firmly absorbed and harder to recover, so more trials should be carried out. This is also reaffirmed with the DFT values obtained, with SO_2_ being more stable. Changing the solvent for a more sustainable one is also a desirable objective and could probably improve our results of the desorption process, generating a better understanding of how others factors work for this kind of material. Another solvent may allow one to expand the working temperature range without losing the interest of maintaining it near room temperature. The proposal of a feasible, renewable, and non-pollutant system for CO_2_ and SO_2_ capture is the final objective of all of our studies.

## 4. Conclusions

This study is a contribution to the theoretical modeling and characterization of porous liquids. It analyzed the feasibility of using a Type II porous liquid for the encapsulation of greenhouse gases through adsorption. The selectivity of the PL was greater towards CO_2_ than SO_2_. However, we observed an increasing trend of SO_2_ adsorption as the temperature increased. Although the CO_2_ occupation rates were higher, we observed that SO_2_ absorption was favored in the later stages of the simulation. DFT calculations showed a greater stability of the C-111/SO_2_ complex vs. the CO_2_ one, suggesting a thermodynamical preference for absorption by SO_2_ and a kinetic preference for CO_2_, in consonance with the MD simulation results. Possible reasons for this preference were also discussed, pointing out the relevance of the chemical properties of the gases.

These results highlight porous liquids as a potential alternative for the development of innovative strategies for greenhouse gas capture and separation due to their intrinsic porosity and high selectivity towards some of these gases. The possibility of using them as a renewable option for the capture of these gases opens an alternative path to mitigate their harmful environmental impact.

## Data Availability

Data are contained within the article.

## Abbreviations

The following abbreviations are used throughout this manuscript:

ATB	Automatic Topology Builder
DCM	dichloromethane
C-111	cryptophane-111
MOF	Metal Organic Framework
PDB	Protein Data Bank
PL	porous liquid
